# IgA Complexes Induce Neutrophil Extracellular Trap Formation More Potently Than IgG Complexes

**DOI:** 10.3389/fimmu.2021.761816

**Published:** 2022-01-13

**Authors:** Anna-Katharina Gimpel, Antonio Maccataio, Harald Unterweger, Maria V. Sokolova, Georg Schett, Ulrike Steffen

**Affiliations:** ^1^ Department of Internal Medicine 3, Friedrich-Alexander-University Erlangen-Nürnberg and Universitätsklinikum Erlangen, Erlangen, Germany; ^2^ Deutsches Zentrum für Immuntherapie, Friedrich-Alexander-University Erlangen-Nürnberg and Universitätsklinikum Erlangen, Erlangen, Germany; ^3^ Department of Otorhinolaryngology, Head and Neck Surgery, Section of Experimental Oncology and Nanomedicine (SEON), Else Kröner-Fresenius-Stiftung Professorship, Friedrich-Alexander-University Erlangen-Nürnberg and Universitätsklinikum Erlangen, Erlangen, Germany

**Keywords:** IgA, IgG, immune complex, NET, neutrophil

## Abstract

Neutrophil extracellular trap (NET) formation is a powerful instrument to fight pathogens, but may induce collateral damage in the affected tissues. Besides pathogen-derived factors, immune complexes are potent inducers of NET formation. Neutrophils express IgA and IgG specific Fc receptors (FcRs) and therefore respond to complexed IgA and IgG. Especially in the context of autoimmune diseases, IgA and IgG immune complexes have been shown to trigger NET formation, a process that putatively contributes to disease severity. However, it is of question if both antibody classes stimulate neutrophils to the same extent. In this study, we compared the capability of IgA and IgG complexes formed by heat aggregation to induce NET formation. While stimulation of neutrophils with IgA complexes robustly induced NET formation, complexed IgG only marginally increased the amount of NETs compared to the unstimulated control. Mixing IgA with IgG before heat aggregation did not increase the effect of complexed IgA on neutrophils. By contrast, the presence of IgG complexes seemed to disturb neutrophil stimulation by IgA complexes. The capacity of complexed IgG to induce NET formation could not be increased by the addition of autologous serum or the removal of terminal sialic acid in the Fc glycan. Together, our data show that IgA is a much more potent inducer of NET formation than IgG. IgA may thus be the main driving force in (auto)immune complex-mediated NET formation.

## Introduction

When tissue is damaged, neutrophils are typically the first immune cells to arrive. They possess several mechanisms to kill invading pathogens and thus represent a powerful part of our body’s first line of defense. Besides the oxidative burst and degranulation, neutrophils are able to release their DNA, a process called neutrophil extracellular trap (NET) formation (1). Due to their stickiness, NETs entrap pathogens and thereby prevent their spreading. In addition, DNA-bound proteases and antimicrobial peptides kill pathogens (2). On the other hand, NETs can cause collateral damage in the affected tissue. NETs are believed to contribute to disease severity in a variety of autoimmune diseases, such as systemic lupus erythematosus (SLE) and rheumatoid arthritis (RA) (3–5). Also in coronavirus-induced disease 2019 (COVID-19), massive NET formation has been shown to aggravate inflammation for example by occluding small blood vessels in the lungs and other organs (6, 7). It is thus very important to understand the trigger mechanisms of NET formation.

Besides various pathogen and damage associated patterns (PAMPs and DAMPs), immune complexes constitute an important trigger of NET formation. Especially in the context of autoimmune diseases, there are several studies showing that immunoglobulin (Ig)A or IgG containing immune complexes induce NET formation (8–11). Human neutrophils not only express several Fc gamma receptors (FγcR), such as as FcγRI, FcγRIIA and FcγRIIIB, but also the FcR for IgA, FcαRI, and can therefore be activated by both IgG and IgA complexes (12, 13). However, the effectiveness of the two Ig classes in this context has not been compared so far. For other neutrophil effector functions, such as antibody-dependent cellular cytotoxicity, it is well described that IgA is a more potent neutrophil stimulator compared to IgG (14) and a similar case is conceivable for NET formation. The individual contribution of single Ig classes to NET formation is an important piece of information in order to better understand their impact on the defense against pathogens, but also on the development of autoimmune diseases. The situation is further complicated by the fact that, in most diseases, (auto)antibodies of the IgA and the IgG class can be found in the patients’ sera (15, 16). It is thus likely that in most conditions mixed immune complexes containing both Ig classes are present. Such mixed immune complexes may elicit stronger responses as they are able to simultaneously activate different FcR types. However, this has not been investigated so far.

To shed light on these questions, we compared the capability of complexed IgA and IgG to induce NET formation in the present study. In addition, we formed mixed complexes to investigate whether IgA and IgG potentiate each other’s effects.

## Methods

### Isolation of IgA and IgG

IgA and IgG were purified from pooled serum of healthy donors. The study was approved by the Ethical Committee of Friedrich-Alexander-Universität Erlangen-Nürnberg. All individuals were informed and agreed to participate in the study.

Total serum IgA was isolated using peptide-M agarose (#gel-pdm-2; *In vivo*gen) according to the manufacturer’s instruction. After three washing steps with phosphate-buffered saline (PBS, pH 7.2) and one washing step with 0.2 M glycine (pH 5) to remove all unspecifically bound proteins, IgA was eluted with 0.1 M glycine (pH 2.7) and immediately neutralized with 1 M tris(hydroxymethyl)aminomethane (Tris)-HCl (pH 9). The eluate was concentrated and rebuffered from elution buffer to PBS with Amicon Ultra Centrifugal Filters (#UFC905024; Merck) according to the manufacturer’s protocol. IgA was then further purified with jacalin agarose (#20395; Thermo Scientific™) according to the manufacturer’s instruction. IgA bound to the agarose was eluted using 0.1 M galactose buffer, concentrated and rebuffered to PBS using Amicon Ultra Centrifugal Filters (#UFC905024; Merck).

IgG was isolated from the IgA depleted peptide-M agarose flow-through using a protein G column (#GE28-9852-55; GE-Healthcare) according to the manufacturer’s instruction. Bound IgG was eluted, neutralized, concentrated and buffered to PBS as described for IgA.

Both IgA and IgG were treated with Triton X-114 (#1001253515; Sigma) to eliminate possible traces of endotoxins. Triton X-114 is a non-ionic detergent that dissolves in aqueous solutions at low temperature and entraps LPS in micelles when heated up again to temperatures above 22°C (17). Antibodies were incubated for 10 minutes with 1% Triton X-114 on ice. This was followed by a 10 minutes incubation step at 37°C which leaded to the separation of TritonX-114 and the aqueous phase. The Triton X-114 phase and the aqueous phase were separated by centrifugation for 10 minutes at 20 000g and room temperature. The aqueous phase containing the antibodies was then carefully transferred into a fresh reaction tube. This procedure was repeated two times. Remaining TritonX-114 in IgA and IgG fractions was removed using Pierce™ detergent removal spin columns (#87778; Thermo Scientific™). Protein concentration was determined with the DC protein assay (#5000111; Bio-Rad).

### Confirmation of IgA and IgG Purity by Western Blot

0.1 µg of isolated IgA or IgG were loaded on a 10% sodium dodecyl sulfate (SDS)–polyacrylamide gel under reducing conditions (5 minutes boiling at 95°C in the presence of β-mercaptoethanol). As standard, Page Ruler™ Plus Prestained Protein Ladder (#26619; Thermo Scientific™) was used. After transfer to a nitrocellulose membrane (#162-0112; BIO-RAD), the membrane was blocked with 5% milk powder in Tris-buffered saline with 0.1% Tween 20 (TBST) and incubated overnight with horseradish peroxidase (HRP)-conjugated antibodies against the proteins of interest (IgA: 1:400; #9130-05, IgG: 1:10000; #2040-05, Southern Biotech). Detection was performed with chemiluminescence reagent (Pierce™ ECL; #32106; Thermo Scientific™) on a chemiluminescence-imager (Celvin S Chemiluminescence Imaging, Biostep).

### Generation, Validation and Size Measurement of Antibody Complexes

IgA and IgG were heat aggregated for 30 minutes at 63°C at a concentration of 5 mg/ml in PBS. Mixed complexes were created by adding IgA and IgG in different ratios (1:1, 1:4) to a total concentration of 5 mg/ml followed by heat aggregation for 30 minutes at 63°C. For the investigation of the effect of complex size on NET formation, IgA and IgG were heat aggregated at concentrations of 2 to 20 mg/ml.

Formation of IgA-IgG mixed complexes was tested with ELISA analysis using capture antibodies against IgA (1:200; #2052-01; SouthernBiotech) and IgG (1:350; #709-006-149; Jackson Immuno Research) and HRP-conjugated detection antibodies against IgA (1:32000; #2050-05) and IgG (1:32000; #2040-05; both SouthernBiotech). Optical density (OD) was measured at 450 nm with a reference wavelength of 620 nm using Sunrise-Basic-Tecan (Tecan).

Complex size was analyzed *via* dynamic light scattering at 25°C using a Zetasizer Nano ZS (Malvern Panalytical) in backscattered mode (173°). IgA, IgG or mixed complexes were aggregated as described above at a concentration of 5 mg/ml and diluted 1:35 in PBS prior to the measurement.

### Neutrophil Isolation and NET Formation Assays

Blood was taken from male and female healthy donors without any medication (age-range 22-46 years) with lithium heparin tubes and neutrophils were immediately isolated by standard Ficoll density gradient centrifugation (Lymphoflot; #824012; BioRad). After taking the polymorph nuclear cell (PMN) fraction, hypotonic lysis of erythrocytes was performed two times by incubation with 36 ml of sterile water for 20 s followed by immediate restoration of tonicity with 4 ml of 10x PBS. After one washing step with PBS, neutrophils were resuspended in RPMI medium without phenol red (#11835-063; Gibco) and supplemented with 1% penicillin/streptomycin (#15140-122; Gibco) and 1% glutamine (#25030-024; Gibco). A sterile 96-well cell culture plate (#644180; Greiner) was pre-incubated for one hour at 37°C and 5% CO_2_ with 155 µl RPMI plus Sytox Green (1:1200; #S7020; Thermo Scientific™) and the corresponding stimuli human serum albumin (HSA) (#126658-1GM; Sigma), heat-aggregated IgA (HAA), heat-aggregated IgG (HAG) or mixed complexes (all 150 µg/ml). As positive control for NET formation, 10 nM of Phorbol 12-myristate 13-acetate (PMA; Sigma) was used. Blockage of NET formation was achieved by adding 10 µM of the neutrophil NADPH oxidase inhibitor diphenylene iodonium (DPI; Sigma).

For Fc-block assays, antibodies against FcαRI (#MCA1824EL; BioRad), FcγRI (clone 10.1; #305047; Biolegend), FcγRII (#GTX74628; Genetex), FcγRIII (clone 3G8; #302050; Biolegend) or isotype control (clone MG1-45; #401408; Biolegend) (all 10 µg/ml) were applied additionally to the stimuli.

For experiments with autologous serum, an additional serum tube was taken from the neutrophil donor. During preincubation of the plate, 2% autologous serum was added to the stimuli.

After preincubation of the plate, neutrophils were seeded into each well in a final concentration of 750000 cells/ml (150000 cells/well in a final volume of 200 µl) and NET-formation assay was performed for 4½ hours at 37°C and 5% CO_2_. Released NETs containing DNA were bound by Sytox Green and the resulting increase in fluorescence was detected every 15 minutes with the fluorescence reader Tecan infinite 200 pro (Tecan). Excitation wavelength was determined at 488 nm, emission wavelength was set at 535 nm. For analysis of NET formation, the average fluorescence values of duplicates were normalized on the average fluorescence value from the last time point of the respective control.

### Visualization of Formed NETs

For live visualization of NET formation, neutrophils were isolated and stimulated as described above using a black 96 well plate (#89626; ibidi) for video analysis. After stimulation, PMNs were allowed to settle for 45 minutes in a live cell imaging chamber of a BZ-X710 microscope (Keyence) at 37°C and 5% CO_2_ before pictures were taken. Pictures were taken every 15 minutes over four hours using a brightfield channel for visualization of neutrophils and a fluorescence channel for visualization of Sytox Green (excitation: 470/40 nm, emission 525/50 nm).

For immunofluorescence staining of NETs, neutrophils were isolated and stimulated in chamber slides (#177445; Thermo Scientific™) as described above, but without Sytox Green. Neutrophils were incubated for 4½ hours at 37°C and 5% CO_2_ after stimulation. During subsequent fixation with 4% paraformaldehyde (PFA) over 20 minutes, the chamber slides were centrifuged for 10 minutes with 800 g at room temperature to fix NETs and neutrophils on the chamber slides. After washing with PBS, permeation was performed with 0.1% Triton X-100 for 8 minutes followed by blocking with 2% BSA in PBS for one hour. Incubation with a primary antibody against neutrophil elastase (1:1000; # PA5-87158; Invitrogen) was performed overnight at 4°C. Secondary Alexa Fluor 647-coupled donkey anti-rabbit antibody (5 µg/ml; #A32795; Invitrogen) and Sytox Green (1:2000; Thermo Scientific™) were added for 1½ hours. Pictures were taken with THUNDER Imager 3D Cell Culture (Leica, SN:536075) with a HC PL FLUOTAR 20x/0,55 PH2 objective (#11506518; Leica). For detection of Sytox green, an excitation wavelength of 475 nm and an emission filter of 535/70 nm was used. For detection of neutrophil elastase, an excitation wavelength of 635 nm and an emission filter of 647/80 nm was used.

### Viability Assay

For investigation of cell viability, neutrophils were isolated as described above, resuspended in RPMI medium without phenol red that was supplemented with 1% penicillin/streptomycin and 1% glutamine (both Gibco, Invitrogen) and seeded in a 96-well cell culture plate (200 μl with 150,000 cells per well). Neutrophils were first incubated with the indicated stimuli (HSA, HAG, HAA, PMA, DPI) at 37°C and 5% CO2. In addition, some neutrophils were heated for 5 min to 65°C to induce cell death (= positive control). Unstimulated neutrophils served as a negative control. A volume of 20 μl of alamarBlue reagent (Thermo scientific) was added to each well. Viability of the cells was analyzed in an Infinite^®^ 200 PRO plate reader (Tecan) at 37°C and 5% CO2 by the assessment of the absorbance at the wavelengths 570 and 595 nm every hour for a total of 4 h.

### Enzymatic Desialylation of IgG

For desialylation, isolated IgG was incubated with 50 U/mg α2-3,6,8 neuraminidase (#P0720L; New England Biolabs) for 16 hours at 37°C. For neuraminidase removal, IgG was re-purified using a Protein G column as described above, followed by LPS removal.

Digestion of sialic acid was verified by lectin blot analysis. 1 µg of desialylated or untreated IgG were resolved in a 10% SDS–polyacrylamide gel and transferred to a PVDF membrane (#162-0177; Bio-Rad). After blocking with 3% deglycosylated gelatin, blots were incubated overnight with biotinylated sambuccus nigra lectin (2 μg/ml; #B-1305-2; Vector laboratories) for detection of sialic acids, followed by incubation with HRP-labeled streptavidin (1:200; #890803; R&D). Detection was performed with ECL (#32106; Thermo Scientific™) on a chemiluminescence-imager (Celvin S Chemiluminescence Imaging, Biostep). In addition, detection of IgG was performed as described above.

### Statistics

Statistical analysis was performed using GraphPad Prism 9.0.2 software. All data sets were tested for normality with Shapiro-Wilk test. For comparison of two groups with normal distribution, paired two-tailed Student’s t-test was employed. In case of non-normal distribution, groups were compared using Wilcoxon matched-pairs signed rank test. Statistics for three or more groups were calculated with repeated measures one-way analysis of variance (ANOVA) for selected pairs of columns followed by Šídák’s multiple comparisons *post hoc* test with Geisser Greenhouse correction in case of normal distribution and Friedmann test followed by Dunn’s multiple comparisons test in case of non-normal distribution. P values less than 0.05 were considered significant. Data are presented as scatter plots with bars showing mean and standard error of mean (SEM).

## Results

### Complexed IgA Has a Higher Potency to Induce NETs Than Complexed IgG

To investigate the effects of IgA and IgG complexes on NET formation, we isolated IgA and IgG from pooled sera of healthy donors and heat-aggregated them at 63°C. We chose heat aggregation as it is not restricted to certain antigen specificities and avoids putative effects arising from the antigen. IgA and IgG purity was certified by western blot (**Supplementary Figure 1**).

**Figure 1:**
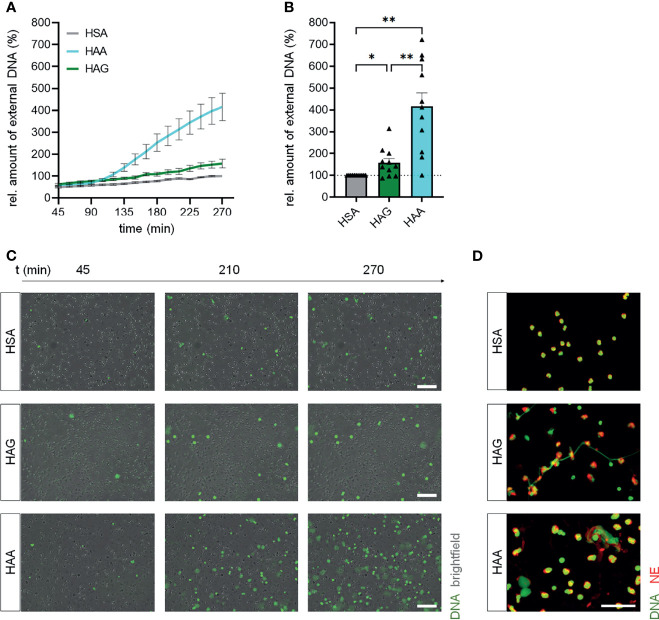
IgA complexes show higher potential to induce NETs than IgG complexes. **(A–C)** Isolated human blood neutrophils were stimulated with 150 µg/ml heat-aggregated IgG (HAG), heat-aggregated IgA (HAA) or human serum albumin (HSA) as control. NET formation was measured by staining extracellular DNA with Sytox Green. **(A)** NET formation over time. **(B)** Relative amount of extracellular DNA 270 min after stimulation normalized on HSA treatment. The dashed line represents the relative amount of NETs of the HSA treated control. N = 11 donors. **(C)** Representative images at 3 different time points. Scale bar = 100 µm. **(D)** Representative images of fixed NETs 270 min after stimulation with HSA, HAG or HAA. Neutrophil elastase (NE) is shown in red, DNA is shown in green. Scale bar = 50 µm. Data are presented as mean ± SEM **(A)** or scatter plots with bars showing mean and SEM **(B)**. Significance was tested with repeated measures one way ANOVA with Geisser Greenhouse correction and Šídák’s multiple comparisons *post hoc* test. *p < 0.05 and **p < 0.01.

Stimulation of isolated blood neutrophils from healthy donors with aggregated IgA led to a robust induction of NET formation compared to the control cells stimulated with HSA (**Figures 1A–C**), although the induction was not as strong as with the potent chemical inducer PMA (**Supplementary Figure 2**). Aggregated IgG also significantly increased NET formation, but the effect was lower than with complexed IgA. No difference in viability was observed for neutrophils treated with aggregated IgA, aggregated IgG, HSA or PMA (**Supplementary Figure 3**).

Staining for neutrophil elastase and DNA confirmed that IgA and IgG complexes induce NET formation (**Figure 1D**). Interestingly, the structure of formed NETs after IgA and IgG stimulation seems to be different. NETs induced by complexed IgG look fibrous, while complexed IgA induced the formation of rather spread out NETs.

### Addition of IgG Does Not Further Increase the NET Forming Capacity of IgA Complexes

As immune complexes in living organisms likely contain a mixture of IgA and IgG, we investigated if the two Ig classes have additive effects on each other regarding the induction of NET formation. We therefore mixed IgA and IgG at various ratios before heat aggregation. The formation of complexes that contained both, IgA and IgG, was confirmed by ELISA using a capture antibody against IgA and a detection antibody against IgG (**Supplementary Figure 4**). As expected, mixed complexes gave high OD signals, while pure IgA or IgG complexes were not detected with this antibody combination.

**Figure 2:**
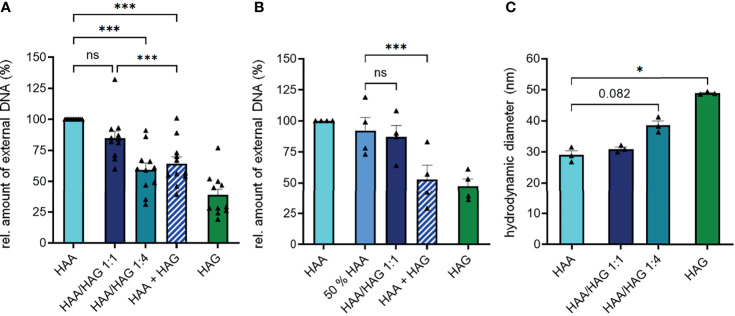
IgG does not increase IgA-induced NET formation in IgA/IgG mixed complexes. **(A)** Isolated human blood neutrophils were stimulated with 150 µg/ml heat-aggregated IgA (HAA), heat-aggregated IgG (HAG), IgA/IgG mixed complexes in different ratios (HAA/HAG 1:1 or 1:4) or 75 µg/ml heat-aggregated IgA plus 75 µg/ml heat-aggregated IgG (HAA + HAG). NET formation was measured by staining extracellular DNA with Sytox Green. Shown are relative amounts of extracellular DNA 270 min after stimulation normalized on HAA treatment. N = 11 donors. **(B)** NET formation of human neutrophils with 150 µg/ml heat-aggregated IgA (HAA), heat-aggregated IgG (HAG), IgA/IgG mixed complexes at 1:1 ratio (HAA/HAG 1:1), 75 µg/ml heat-aggregated IgA (50% HAA) or 75 µg/ml heat-aggregated IgA plus 75 µg/ml heat-aggregated IgG (HAA + HAG). Shown are relative amounts of extracellular DNA 270 min after stimulation normalized on HAA treatment. N = 4 donors. **(C)** Hydrodynamic diameter of Ig complexes measured with dynamic light scattering. N = 3. Data are presented as scatter plots with bars showing mean and SEM. Significance was tested with repeated measures one way ANOVA with Geisser Greenhouse correction and Šídák’s multiple comparisons *post hoc* test. *p < 0.05; and ***p < 0.001. ns, not significant.

In line with the observation that IgG had a lower potential to induce NET formation, mixed IgA/IgG complexes (HAA/HAG) did not result in the formation of more NETs compared to pure IgA complexes (**Figure 2A**). In contrast, a high percentage of IgG (IgA : IgG ratio of 1:4) even significantly decreased IgA-mediated NET formation. Interestingly, a 1:1 combination of separately aggregated pure IgA and IgG complexes (HAA+HAG) even induced less NETs than complexed IgA alone or mixed IgA/IgG complexes at a ratio of 1:1. This phenomenon cannot be explained by the lower IgA concentration in the HAA+HAG condition, as also stimulation with pure IgA complexes at half concentration still resulted in higher NET formation compared to stimulation with a combination of pure IgA and IgG complexes (**Figure 2B**).

To determine if heat aggregation of IgA and IgG results in equally sized Ig complexes, we measured their hydrodynamic diameter with dynamic light scattering. This measurement revealed that IgG formed slightly larger complexes than IgA after heat aggregation (**Figure 2C**), excluding the possibility that IgA complexes show superior NET formation induction because of a larger size. Concomitantly, mixed complexes of IgA and IgG became larger with increasing IgG proportion.

To investigate if immune complex size might influence NET formation, we generated IgA and IgG complexes of different sizes (23 to 35 µm diameter for aggregated IgA and 36 to 91 µm diameter for aggregated IgG) by using different antibody concentrations during heat aggregation (**Supplementary Figure 5**). Especially IgG complexes became much larger when aggregated at a higher concentration. However, the amount of NETs formed after stimulation of neutrophils with the differently sized Ig aggregates was not changed, indicating that the immune complex size is not influencing NET formation.

### FcR Dependency of Ig Complex-Induced NET Formation

**Figure 3:**
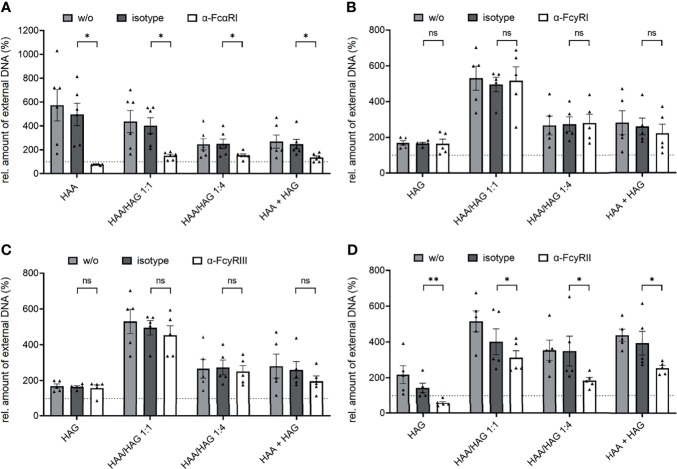
IgA and IgG complex-induced NET formation is dependent on FcαRI and FcγRII, respectively. Isolated human neutrophils were stimulated with 150 µg/ml heat-aggregated IgA (HAA), heat-aggregated IgG (HAG), IgA/IgG mixed complexes in different ratios (HAA/HAG 1:1 or 1:4) or 75 µg/ml heat-aggregated IgA plus 75 µg/ml heat-aggregated IgG (HAA + HAG) in the presence of 10 µg/ml blocking antibodies against FcαRI **(A)**, FcγRI **(B)**, FcγRIII **(C)**, FcγRII **(D)** or the respective isotype control. w/o = no additional antibody. Shown are relative amounts of extracellular DNA 270 min after stimulation normalized on HSA treatment. **(A)** N = 4 donors. **(B–D)** N = 5 donors. Data are presented as scatter plots with bars showing mean and SEM. The dashed line represents the relative amount of NETs of the HSA treated control. Significances between isotype and blocking antibody treatment was tested with Wilcoxon matched-pairs signed rank test **(A–C)** or paired t-test **(D)**. *p < 0.05 and **p < 0.01. ns, not significant.

For a better characterization of Ig complex-induced NET formation, we next examined its dependency on FcRs. As expected, blocking antibodies against FcαRI completely abrogated the induction of NET formation by pure IgA complexes and to a great extent the induction of NET formation by mixed IgA/IgG complexes (**Figure 3A**). This result indicates that IgA is the major driving force for NET formation in mixed complexes. NET induction by IgG complexes was not affected by FcαRI blockade (**Supplementary Figure 6A**). Blockade of FcγRI and FcγRIII did not show any effect (**Figures 3B, C**). By contrast, blocking FcγRII completely abrogated the induction of NET formation by pure IgG complexes and to some extent the induction of NET formation by mixed IgA/IgG complexes (**Figure 3D**). IgA complex-induced NET formation was not affected by blocking of any of the FcγRs (**Supplementary Figure 6B**).

### Autologous Serum Reduces NET Formation

**Figure 4:**
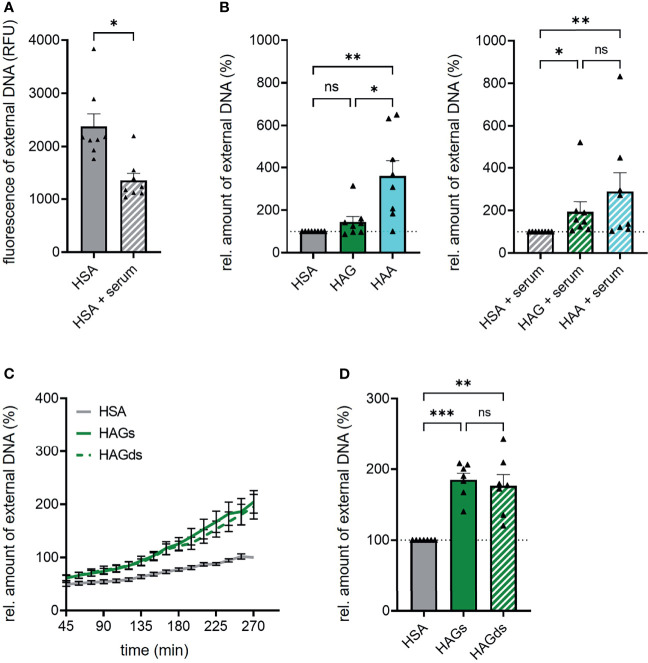
Autologous serum and sialylation status do not influence IgG complex-induced NET formation. **(A)** Spontaneous NET formation of isolated human neutrophils in the presence of 150 µg/ml human serum albumin (HSA) with or without 2% autologous serum. NET formation was measured by staining extracellular DNA with Sytox Green. Shown is fluorescence intensity of Sytox Green 270 min after HSA addition. N = 8 donors. **(B)** NET formation of human neutrophils with 150 µg/ml heat-aggregated IgG (HAG), heat-aggregated IgA (HAA) or human serum albumin (HSA) in the absence or presence of 2% autologous serum. Shown are relative amounts of extracellular DNA 270 min after stimulation normalized on HSA treatment. The dashed line represents the relative amount of NETs of the HSA treated control. N = 8 donors. **(C, D)** NET formation of human neutrophils with 150 µg/ml heat-aggregated native IgG (HAGs), heat-aggregated desialylated IgG (HAGds) or human serum albumin (HSA). **(C)** NET formation over time. **(D)** Relative amounts of extracellular DNA 270 min after stimulation normalized on HSA treatment. The dashed line represents the relative amount of NETs of the HSA treated control. N = 7 donors. Data are presented as mean ± SEM **(C)** or scatter plots with bars showing mean and SEM **(A, B, D)**. Significance was tested with Wilcoxon matched-pairs signed rank test **(A)** repeated measures one way ANOVA with Geisser Greenhouse correction and Šídák’s multiple comparisons *post hoc* test **(D)** or Friedmann test followed by Dunn’s multiple comparisons test **(B)** *post hoc*. *p < 0.05; **p < 0.01; and ***p < 0.001. ns, not significant.

Overall, complexed IgG was surprisingly weak in inducing NET formation in our experimental setting. One possible explanation could be that additional factors are needed for efficient IgG complex-mediated NET formation. Serum-derived complement for example has been shown to increase the capability of IgG complexes to induce NET formation (18). We thus compared NET formation induced by IgA and IgG complexes in the presence of 2% autologous serum. In general, the addition of serum led to a marked reduction of spontaneous NET formation (**Figure 4A**) and in a less pronounced difference between HAA and HAG-induced NET formation that was not significant anymore. This effect mainly seems to be caused by an at least partial block of IgA complex-induced NET formation after serum addition (**Figure 4B**). In contrast, serum seemed to have no or even a small positive effect on the capacity of IgG complexes to induce NET formation.

### IgG Fc Sialylation Does Not Affect NET Formation

Another factor that may have influenced our results is the fact that we used IgA and IgG from healthy donors, whereas in other publications often immune complexes isolated from patients have been used. In patients with chronic inflammatory diseases, total serum IgG as well as antigen-specific autoantibodies display different Fc glycosylation profiles compared to healthy persons, including lower degrees of galactosylation and sialylation (19–21). Especially the lack of terminal sialic acid has been shown to increase inflammatory effector functions of IgG (22). We thus tested if the cleavage of terminal sialic acid increases the capacity of IgG complexes to induce NET formation. Successful desialylation was confirmed with lectin blot analysis (**Supplementary Figure 7**). However, IgG Fc sialylation does not seem to play a role in NET formation, as neutrophils stimulated with native or desialylated IgG complexes showed exactly the same NET formation rate (**Figures 4C, D**).

## Discussion

NETs are associated with tissue destruction in several diseases including SLE, RA and COVID-19 (3–6). In these diseases, immune complexes represent an important trigger of NET formation. It is thus important to better understand the mechanisms driving immune complex-mediated NET formation.

In most diseases, specific antibodies can be found in the form of IgG and IgA and it is very likely that immune complexes are composed of a mixture of these antibody classes. However, it has not been investigated yet if both Ig classes equally contribute to NET formation or if one class constitutes the main driving force.

In the present study, we found IgA to be much more effective in inducing NET formation compared to IgG. This observation goes in line with published data reporting that IgA, but not IgG potently induces neutrophil-mediated tumor cell killing (14, 23). We recently found that in COVID-19, virus-specific IgA, but not IgG is associated with increased NET formation markers (24). NET formation induces severe complications in COVID-19 (6, 7, 25), and in our recent study, virus-specific IgA was associated with a more severe disease course and fatal outcome (24).

There are some studies describing IgA or IgG-driven induction of NET formation (8–11). However, no study has compared the effects of IgA and IgG so far. Interestingly, according to our data, it seems that the presence of IgG complexes even blunted the efficacy of complexed IgA to induce NETs. This effect is difficult to explain, as Fc receptor blockade revealed that IgG actively contributed to NET formation *via* FcγRII. One possible explanation might be steric competition of IgA and IgG, but this is difficult to test.

In our study, we used heat aggregated IgA and IgG to investigate the efficacy of IgA and IgG complexes to induce NET formation. The IgA and IgG aggregates were quite standardized in size, while the size of immune complexes might vary greatly *in vivo*. Unfortunately, information about the exact size of isolated immune complexes is scarce. Velichko et al. described immune complexes in serum to be sized around 40 – 200 nm in diameter (26). This is a bit larger than the size of our heat aggregated complexes. However, we did not see an increase in NET formation when using larger complexes, suggesting that the immune complex size does not substantially affect the NET formation rate.

In contrast to IgA, IgG complexes activate complement very well (27). As activated complement has been shown to induce NET formation (28), binding of complement possibly could increase IgG complex-mediated NET formation. Indeed, in the study by Hair *et al.*, IgG-containing immune complexes alone did not induce NET formation, while complement binding enabled by pre-incubation with serum markedly increased immune complex mediated NET formation (18). In our setting, we did not see a pronounced stimulatory effect of serum on IgG complex-induced NET formation. By contrast, we observed a general inhibitory effect of serum on spontaneous and IgA complex-induced NET formation. This has also been described in the literature (1, 29), although it stands in contrast to some publications in which sera from RA patients have been used (8, 10). From a physiological point of view, it makes sense to suppress NET formation in the circulation to avoid blood vessel occlusion, and serum components would be very well suited to do so. On the other hand, it is possible that alterations in the serum composition in patients with inflammatory diseases overcome these protective mechanisms.

In addition to putative effects of serum, we tested if a lack of terminal sialic acid in the Fc glycan could increase IgG complex-induced NET formation. The presence of terminal sialic acid suppresses inflammatory effector functions of IgG (30–32). We recently found that desialylation of IgA increases its capacity to induce NET formation (33), and thus thought that terminal sialic acid might also suppress the capacity of IgG to induce NET formation. Nevertheless, according to our data, it seems that IgG Fc sialylation does not play a role in IgG complex-driven NET formation. Recently, it has been shown that low IgG Fc fucosylation, as it has been found in patients suffering from severe COVID 19, increases the inflammatory impact of IgG on human macrophages (34, 35). This effect was mostly mediated by FcγRIII that has been described to have a markedly improved affinity to afucosylated IgG (36). Although in our study FcγRIII did not seem to be involved in NET formation, it is possible that defucosylation of IgG could increase the stimulation of neutrophil FcγRIII by IgG complexes to an extend that it starts to contribute to NET formation. However, as the impact of Ig Fc glycosylation on NET formation was not the main focus of this study, we did not investigate the effect of defucosylation or other variations such as degalactosylation on IgA and IgG complex-mediated NET formation.

Altogether, our data strongly suggest that IgA is superior to IgG in inducing NETs. We excluded Ig complex size, serum-derived factors and differences in Fc sialylation to increase the efficacy of IgG complexes to induce NET formation. However, other factors, such as antigen-mediated effects or other glycosylation variants, such as galactosylation or fucosylation could play a role. For future studies involving immune complexes derived from patients’ body fluids, we strongly recommend to look also at IgA as a contributor to NET formation as well as FcαRI blockade to inhibit immune complex mediated NET formation.

## Data Availability Statement

The original contributions presented in the study are included in the article/**Supplementary Material**. Further inquiries can be directed to the corresponding author.

## Ethics Statement

The studies involving human participants were reviewed and approved by the Ethical Committee of Friedrich-Alexander-Universität Erlangen-Nürnberg. The patients/participants provided their written informed consent to participate in this study.

## Author Contributions

A-KG and US: study conception and experimental design. A-KG, AM, and HU: acquisition of data. A-KG, AM, HU, MS and US: data analysis and interpretation. GS and US: funding acquisition. A-KG, and US: drafting the article. A-KG, AM, HU, MS, GS and US: critical revision of the article and final approval of the version to be published. All authors contributed to the article and approved the submitted version.

## Funding

This research was funded by the Deutsche Forschungsgemeinschaft (FOR2886 PANDORA-TP03 and TP04; CRC1181 Resolution of Inflammation), the Interdisciplinary Center for Clinical Research (IZKF) University Clinic Erlangen, the Manfred-Roth-Stiftung, and the European Union (ERC Synergy grant 810316 4D NanoSCOPE and the EU/EFPIA Innovative Medicines Initiative 2 JointUndertaking RTCure grant no. 777357).

## Conflict of Interest

The authors declare that the research was conducted in the absence of any commercial or financial relationships that could be construed as a potential conflict of interest.

## Publisher’s Note

All claims expressed in this article are solely those of the authors and do not necessarily represent those of their affiliated organizations, or those of the publisher, the editors and the reviewers. Any product that may be evaluated in this article, or claim that may be made by its manufacturer, is not guaranteed or endorsed by the publisher.
