# Supplementary material for: IgA Complexes Induce Neutrophil Extracellular Trap Formation More Potently Than IgG Complexes
Source: Front Immunol. 2022 Jan 13;12:761816. doi: 10.3389/fimmu.2021.761816 (PMC8792984; doi:10.3389/fimmu.2021.761816)
Supplement: Supplementary file 1 [file DataSheet_1.pdf]

Supplementary Material

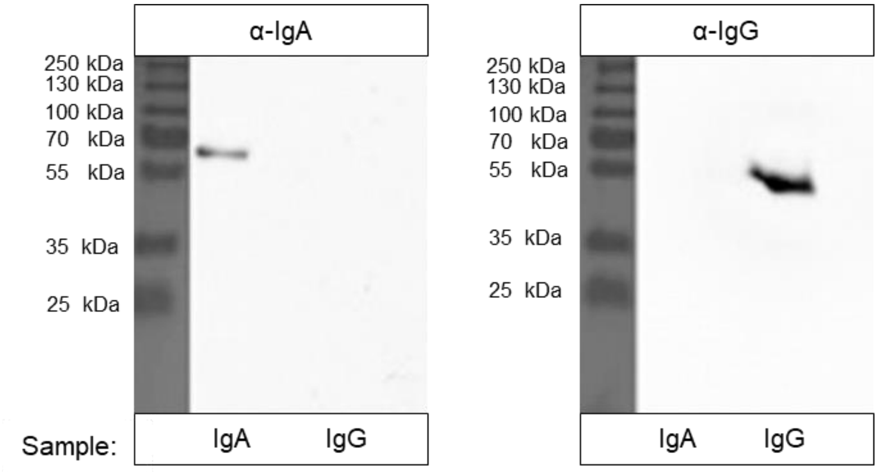

Supplementary Figure 1. Verification of antibody purity. Western blot analysis of isolated IgA and IgG using secondary antibodies against IgA (α-IgA) and IgG (α-IgG).

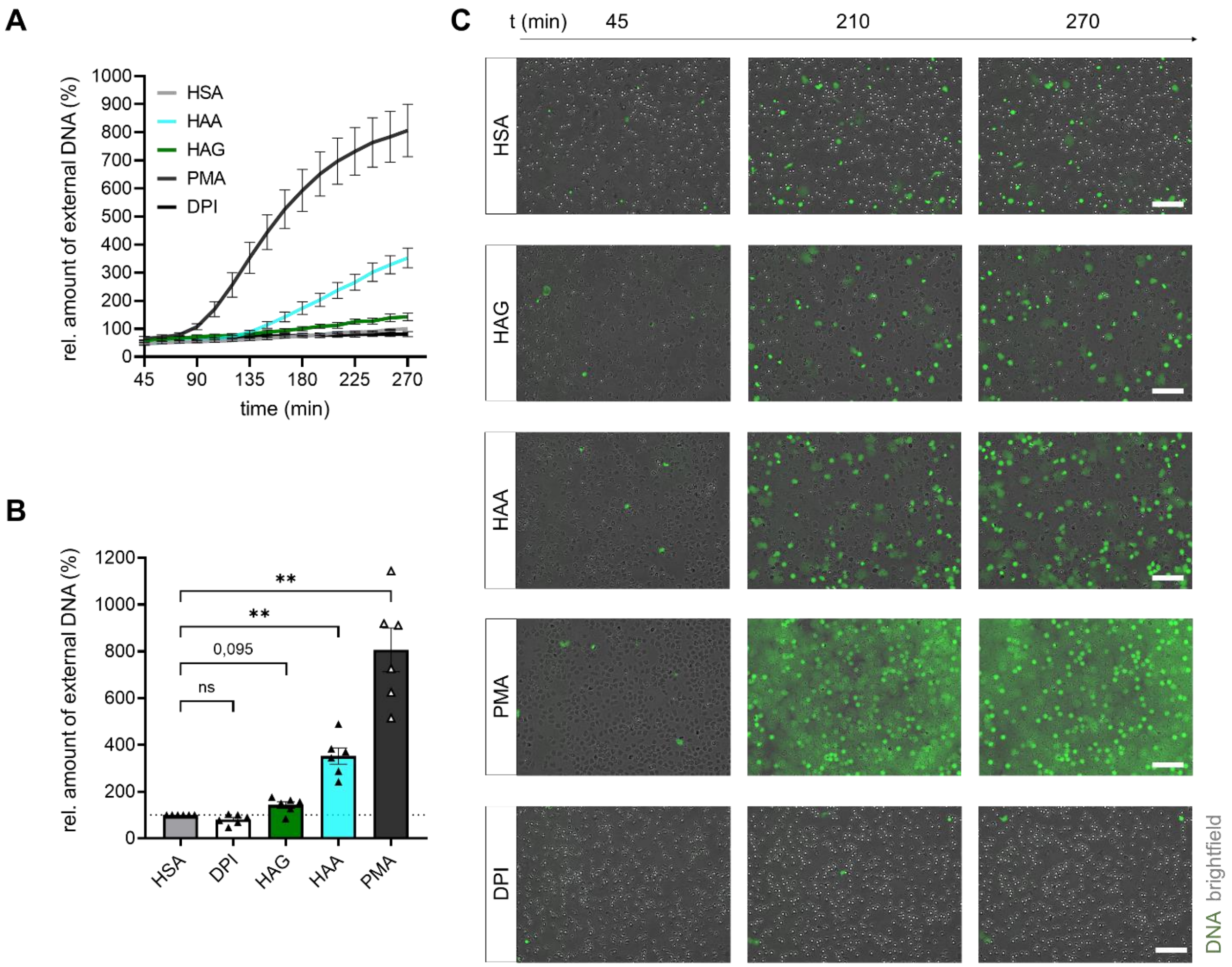

Supplementary Figure 2. Comparison of NET formation after stimulation with HAA, HAG or the chemical activator PMA. Isolated human blood neutrophils were stimulated with 150 µg/ml heat-aggregated IgG (HAG), heat-aggregated IgA (HAA), human serum albumin (HSA), 10 nM of Phorbol 12-myristate 13-acetate (PMA) as positive control or 10 µM of the neutrophil NADPH oxidase inhibitor diphenylene iodonium (DPI) as negative control. NET formation was measured by staining extracellular DNA with Sytox Green. **(A)** NET formation over time. **(B)** Relative amount of extracellular DNA 270 min after stimulation normalized on HSA treatment. The dashed line represents the relative amount of NETs of the HSA treated control. N = 6 donors. **(C)** Representative images at 3 different time points. Scale bar = 100 µm. Data are presented as mean ± SEM **(A)** or scatter plots with bars showing mean and SEM **(B)**. Significance was tested with repeated measures one way ANOVA with Geisser Greenhouse correction and Šídák's multiple comparisons post hoc test. *p < 0.05; **p < 0.01; and ***p < 0.001.

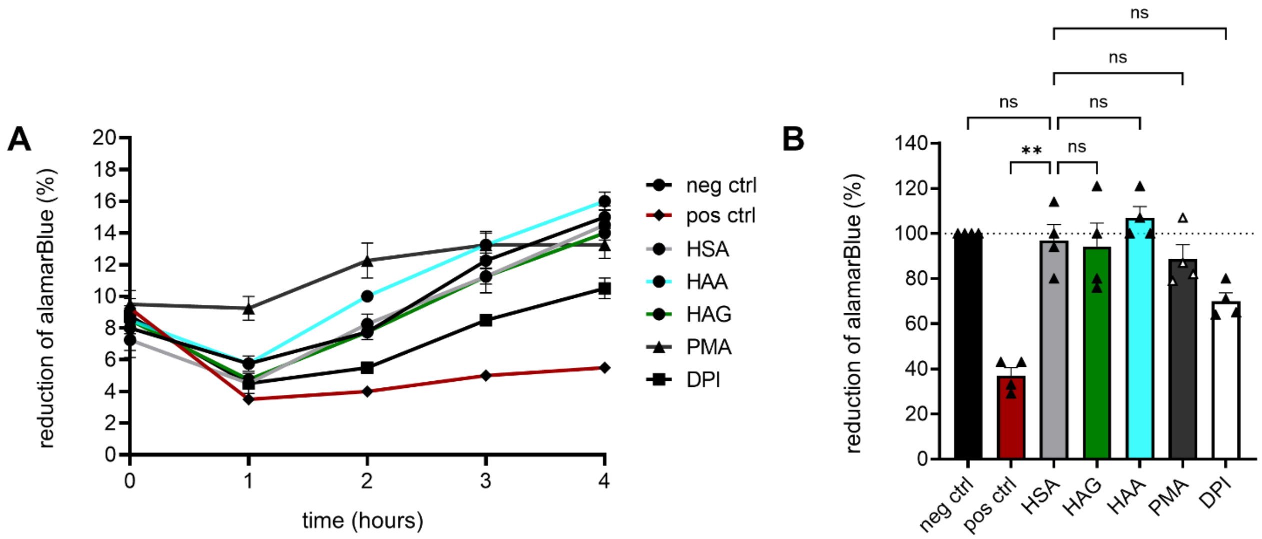

Supplementary Figure 3. Cell viability after stimulation with HAA, HAG or the chemical activator PMA. Isolated human blood neutrophils were stimulated with 150 µg/ml heat-aggregated IgG (HAG), heat-aggregated IgA (HAA), human serum albumin (HSA), 10 nM of Phorbol 12-myristate 13-acetate (PMA) or 10 µM of the neutrophil NADPH oxidase inhibitor diphenylene iodonium (DPI). Cell viability was measured with alamarBlue. **(A)** Reduction of alamarBlue over time. **(B)** Reduction of alamarBlue 4 h after stimulation. N = 4 donors. Data are presented as mean ± SEM **(A)** or scatter plots with bars showing mean and SEM **(B)**. Significance was tested with repeated measures one way ANOVA with Geisser Greenhouse correction and Šídák's multiple comparisons post hoc test. *p < 0.05; **p < 0.01; and ***p < 0.001.

Sample / Cap/Det	HAA	HAA/HAG 4:1	HAA/HAG 1:1	HAA/HAG 1:4	HAG
α-IgG/α-IgG	0.0045	0.627	1.0905	1.3965	1.644
α-IgA/α-IgG	0.018	0.487	0.7775	1.014	0.039
α-IgA/α-IgA	0.3895	0.3485	0.2475	0.091	-0.0005

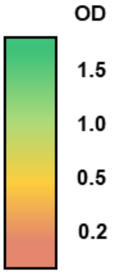

Supplementary Figure 4. Verification of mixed complex formation. Validation of IgA-IgG-complex formation using ELISA. Plates were coated with capture antibodies against IgA (α-IgA) or IgG (α-IgG). Detection was performed with secondary antibodies against IgA (α-IgA) or IgG (α-IgG). HAA = heat aggregated IgA, HAG = heat aggregated IgG, HAA/HAG = complexes of mixed IgA and IgG in different ratios (IgA:IgG = 4:1, 1:1 or 1:4), OD = optical density.

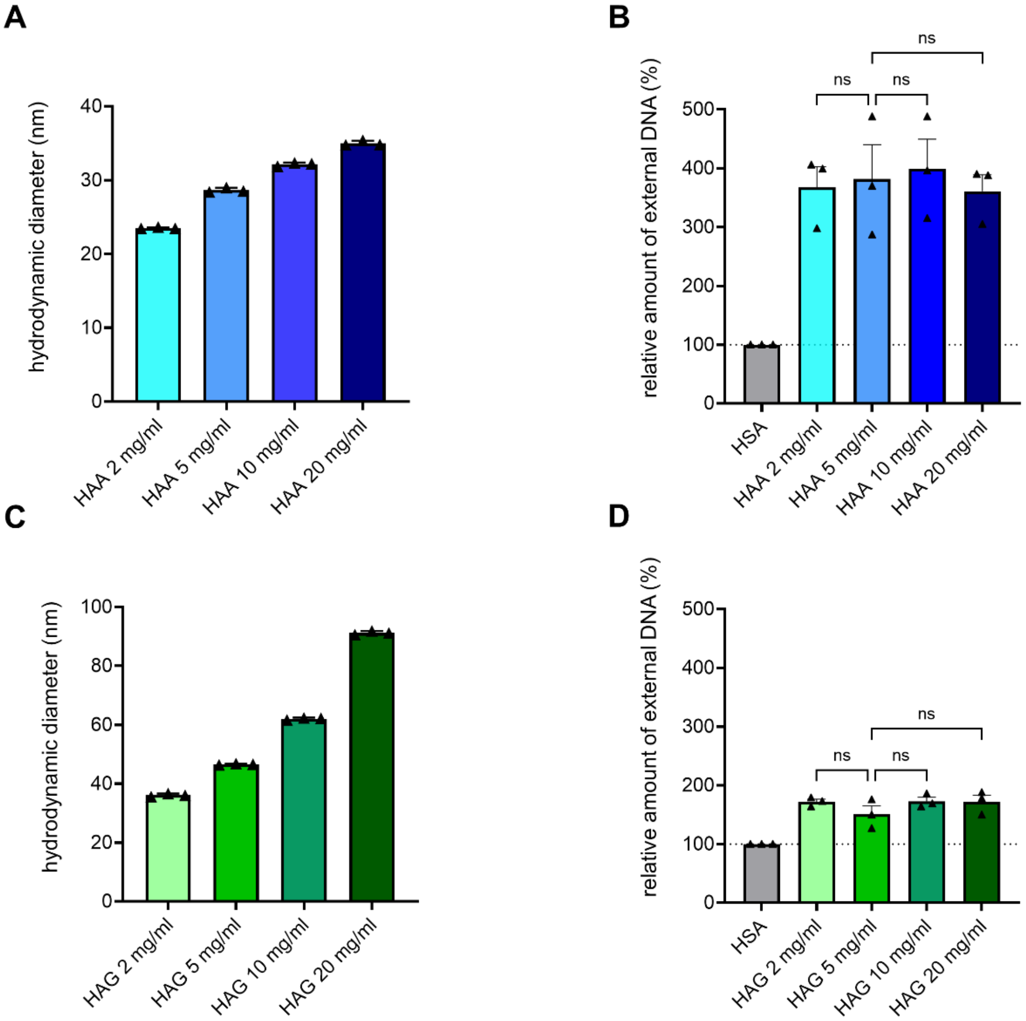

Supplementary Figure 5. Size of IgA and IgG complexes does not affect NET formation rate. (A) Hydrodynamic diameter of IgA complexes aggregated at the indicated concentrations. N = 3. **(B)** NET formation of human neutrophils with 150 µg/ml IgA complexes aggregated at the indicated concentrations. Shown are relative amounts of extracellular DNA 270 min after stimulation normalized on HSA treatment. The dashed line represents the relative amount of NETs of the HSA treated control. N = 3 donors. **(C)** Hydrodynamic diameter of IgG complexes aggregated at the indicated concentrations. N = 3. **(D)** NET formation of human neutrophils with 150 µg/ml IgG complexes aggregated at the indicated concentrations. Shown are relative amounts of extracellular DNA 270 min after stimulation normalized on HSA treatment. The dashed line represents the relative amount of NETs of the HSA treated control. N = 3 donors. Data are presented as scatter plots with bars showing mean and SEM. Significance was tested with Friedmann test followed by Dunn's multiple comparisons test. ns = not significant.

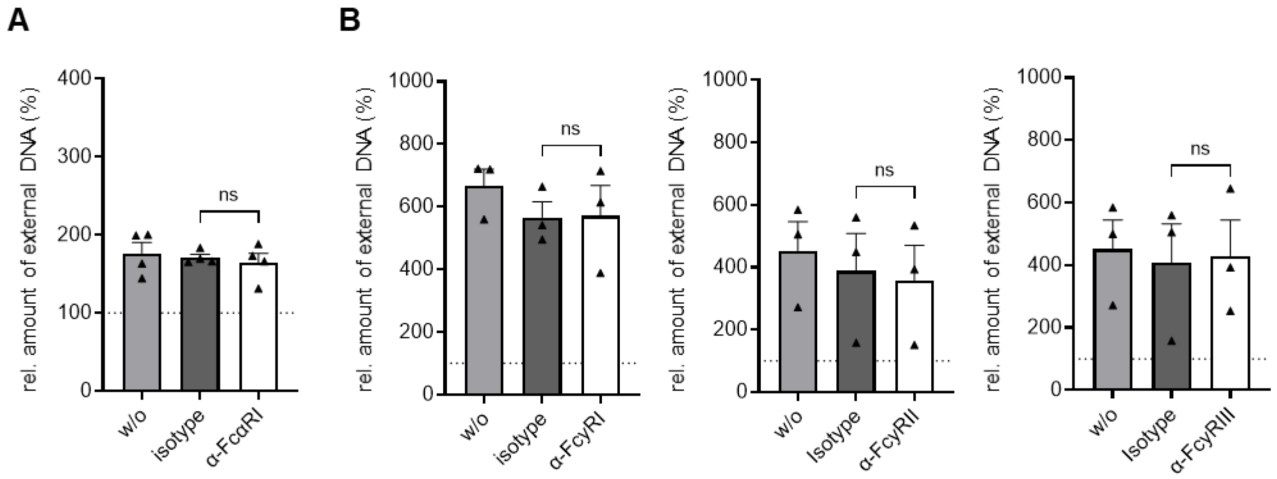

Supplementary Figure 6. FcαRI-block shows no effect on IgG-induced NET-formation and FcγR-block does not affect IgA-induced NET-formation. Isolated human blood neutrophils were stimulated with 150 µg/ml heat aggregated IgG (HAG) **(A)** or heat aggregated IgA (HAA) **(B)** in the presence of 10 µg/ml antibodies against FcαRI, FcγRI, FcγRII, FcγRIII, or the respective isotype control. w/o = no additional antibody. Formation of neutrophil extracellular traps (NET) was analyzed by staining extracellular DNA with Sytox Green. Shown are relative amounts of extracellular DNA 270 min after stimulation normalized on HSA treatment. **(A)** N = 4 donors. **(B)** N = 3 donors. Data are presented as scatter plots with bars showing mean and SEM. The dashed line represents the relative amount of NETs of the HSA treated control. Significances between isotype and blocking antibody treatment was tested with paired t-test (B) or Wilcoxon matched-pairs signed rank test (A, C, D). ns = not significant.

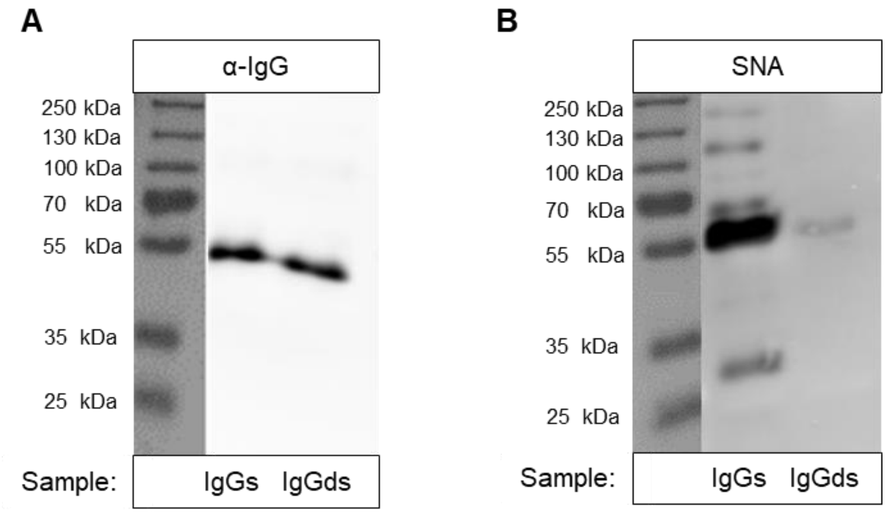

Supplementary Figure 7. Verification of IgG desialylation. Western and lectin blot of native IgG (IgGs) and desialylated IgG (IgGds) using **(A)** a secondary antibody against IgG (α-IgG) as loading control and **(B)** *sambuccus nigra agglutintin* (SNA) to detect terminal sialic acid in the Fc glycan.
